# Carbapenemase-Encoding Gene Copy Number Estimator (CCNE): a Tool for Carbapenemase Gene Copy Number Estimation

**DOI:** 10.1128/spectrum.01000-22

**Published:** 2022-07-05

**Authors:** Jianping Jiang, Liang Chen, Xin Chen, Pei Li, Xiaogang Xu, Vance G. Fowler, David van Duin, Minggui Wang

**Affiliations:** a Institute of Antibiotics, Huashan Hospital, Fudan University, Shanghai, China; b Center for Discovery and Innovation, Hackensack Meridian Health, Nutley, New Jersey, USA; c Department of Medical Sciences, Hackensack Meridian School of Medicine, Nutley, New Jersey, USA; d Division of Infectious Diseases, University of North Carolina, Chapel Hill, North Carolina, USA; e Duke Clinical Research Institute, Duke University Medical Center, Durham, North Carolina, USA; University of Greifswald

**Keywords:** carbapenem resistance, carbapenemase-encoding gene, *Enterobacterales*, gene copy number

## Abstract

Carbapenemase production is one of the leading mechanisms of carbapenem resistance in Gram-negative bacteria. An increase in carbapenemase gene (*bla*Carb) copies is an important mechanism of carbapenem resistance. No currently available bioinformatics tools allow for reliable detection and reporting of carbapenemase gene copy numbers. Here, we describe the carbapenemase-encoding gene copy number estimator (CCNE), a ready-to-use bioinformatics tool that was developed to estimate *bla*Carb copy numbers from whole-genome sequencing data. Its performance on *Klebsiella pneumoniae* carbapenemase gene (*bla*_KPC_) copy number estimation was evaluated by simulation and quantitative PCR (qPCR), and the results were compared with available algorithms. CCNE has two components, CCNE-acc and CCNE-fast. CCNE-acc detects *bla*Carb copy number in a comprehensive and high-accuracy way, while CCNE-fast rapidly screens *bla*Carb copy numbers. CCNE-acc achieved the best accuracy (100%) and the lowest root mean squared error (RMSE; 0.07) in simulated noise data sets, compared to the assembly-based method (23.4% accuracy, 1.697 RMSE) and the OrthologsBased method (78.9% accuracy, 0.395 RMSE). In the qPCR validation, a high consistency was observed between the *bla*_KPC_ copy number determined by qPCR and that determined with CCNE. Reverse transcription-qPCR transcriptional analysis of 40 isolates showed that *bla*_KPC_ expression was positively correlated with the *bla*_KPC_ copy numbers detected by CCNE (*P* < 0.001). An association study of 357 KPC-producing K. pneumoniae isolates and their antimicrobial susceptibility identified a significant association between the estimated *bla*_KPC_ copy number and MICs of imipenem (*P* < 0.001) and ceftazidime-avibactam (*P* < 0.001). Overall, CCNE is a useful genomic tool for the analysis of antimicrobial resistance genes copy number; it is available at https://github.com/biojiang/ccne.

**IMPORTANCE** Globally disseminated carbapenem-resistant *Enterobacterales* is an urgent threat to public health. The most common carbapenem resistance mechanism is the production of carbapenemases. Carbapenemase-producing isolates often exhibit a wide range of carbapenem MICs. Higher carbapenem MICs have been associated with treatment failure. The increase of carbapenemase gene (*bla*Carb) copy numbers contributes to increased carbapenem MICs. However, *bla*Carb gene copy number detection is not routinely conducted during a genomic analysis, in part due to the lack of optimal bioinformatics tools. In this study, we describe a ready-to-use tool we developed and designated the carbapenemase-encoding gene copy number estimator (CCNE) that can be used to estimate the *bla*Carb copy number directly from whole-genome sequencing data, and we extended the data to support the analysis of all known *bla*Carb genes and some other antimicrobial resistance genes. Furthermore, CCNE can be used to interrogate the correlations between genotypes and susceptibility phenotypes and to improve our understanding of antimicrobial resistance mechanisms.

## INTRODUCTION

The worldwide spread of carbapenem-resistant *Enterobacterales* (CRE) is an urgent public health threat (1). The most common carbapenem resistance mechanism is the production of carbapenemases. Among them, the Klebsiella pneumoniae carbapenemase (KPC) is the most prevalent type in CRE (2, 3). Other carbapenemases, including New Delhi metallo-β-lactamase (NDM), some oxacillin carbapenemases (OXA), Verona integron-encoded metallo-β-lactamase (VIM), and imipenemase (IMP), are also frequently detected in *Enterobacterales* (4). Carbapenemases may also be found in other Gram-negative bacilli, such as Pseudomonas and Acinetobacter species (5).

Carbapenemase-producing isolates often exhibit a wide range of carbapenem MICs. Higher carbapenem MICs have been associated with treatment failure (3, 6). It has been reported that the increase of carbapenemase gene (*bla*Carb) copy number contributes to increased carbapenem MICs (7), as well as to resistance to novel β-lactam and β-lactamase inhibitors, such as ceftazidime-avibactam (CAZ-AVI) (8), meropenem-vaborbactam (9), and imipenem-relebactam. However, *bla*Carb gene copy number detection is not routinely conducted during a genomic analysis, in part due to the lack of optimal bioinformatics tools. Even though some studies have examined the impact of *bla*Carb copy number variations on antimicrobial susceptibility changes, most of these studies were limited by small sample size (10–12).

Tools such as CNVnator (13), CNV-BAC (14), and CNOGpro (15) have been developed to analyze gene copy number from whole-genome sequencing (WGS) data. However, they are not optimal for *bla*Carb gene copy number detection, because *bla*Carb is often located on a plasmid with high sequence plasticity. Methods including AssemblyBased (16, 17) and OrthologsBased approaches (18) were developed to analyze the copy number of *bla*_KPC_ in K. pneumoniae, Escherichia coli, and Pseudomonas aeruginosa by using WGS data. However, their accuracies on gene copy number estimation have not been fully evaluated, and no ready-to-use tools are available.

In this study, we developed a ready-to-use tool designated the carbapenemase-encoding gene copy number estimator (CCNE) to estimate the *bla*Carb copy number directly from WGS data, and we extended our data to support the analysis of all known *bla*Carbs and some other antimicrobial resistance genes. CCNE has two components, CCNE-acc and CCNE-fast. CCNE-acc detects *bla*Carb copy number in a comprehensive and high-accuracy way and uses the assembled contigs to estimate *bla*Carb copy number, without species limitations. CCNE-fast is designed for rapid screening of *bla*Carb copy number. CCNE uses a reference gene to estimate *bla*Carb copy number and it has now supported the analysis of 111 species. We evaluated the performance of CCNE-acc and CCNE-fast on *bla*_KPC_ copy number estimation both by simulation and through analyses of real data. We compared the results with those from AssemblyBased and OrthologsBased methods. Further, we applied CCNE to analyze the *bla*_KPC_ copy numbers of 357 KPC-producing K. pneumoniae clinical isolates and correlated the results with imipenem and ceftazidime-avibactam MICs.

## RESULTS

### Implementation of CCNE.

**FIG 1:**
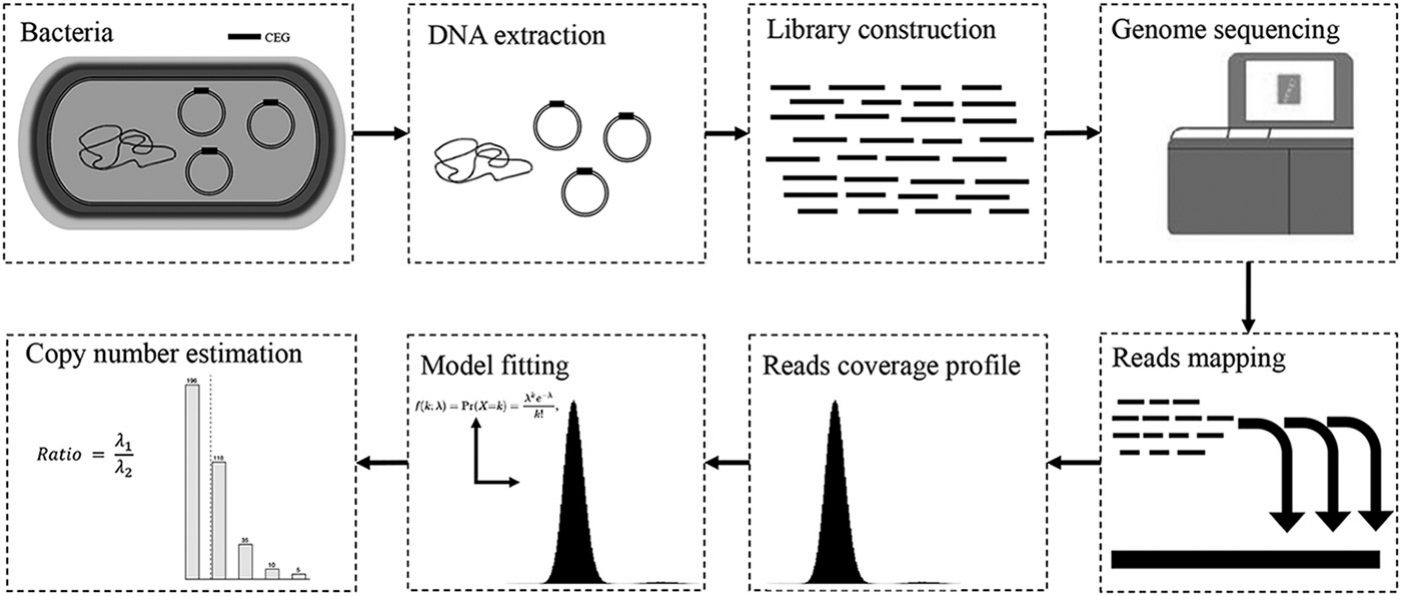
The general procedure of determining the *bla*Carb copy number by read depth-based methods from WGS reads.

**FIG 2:**
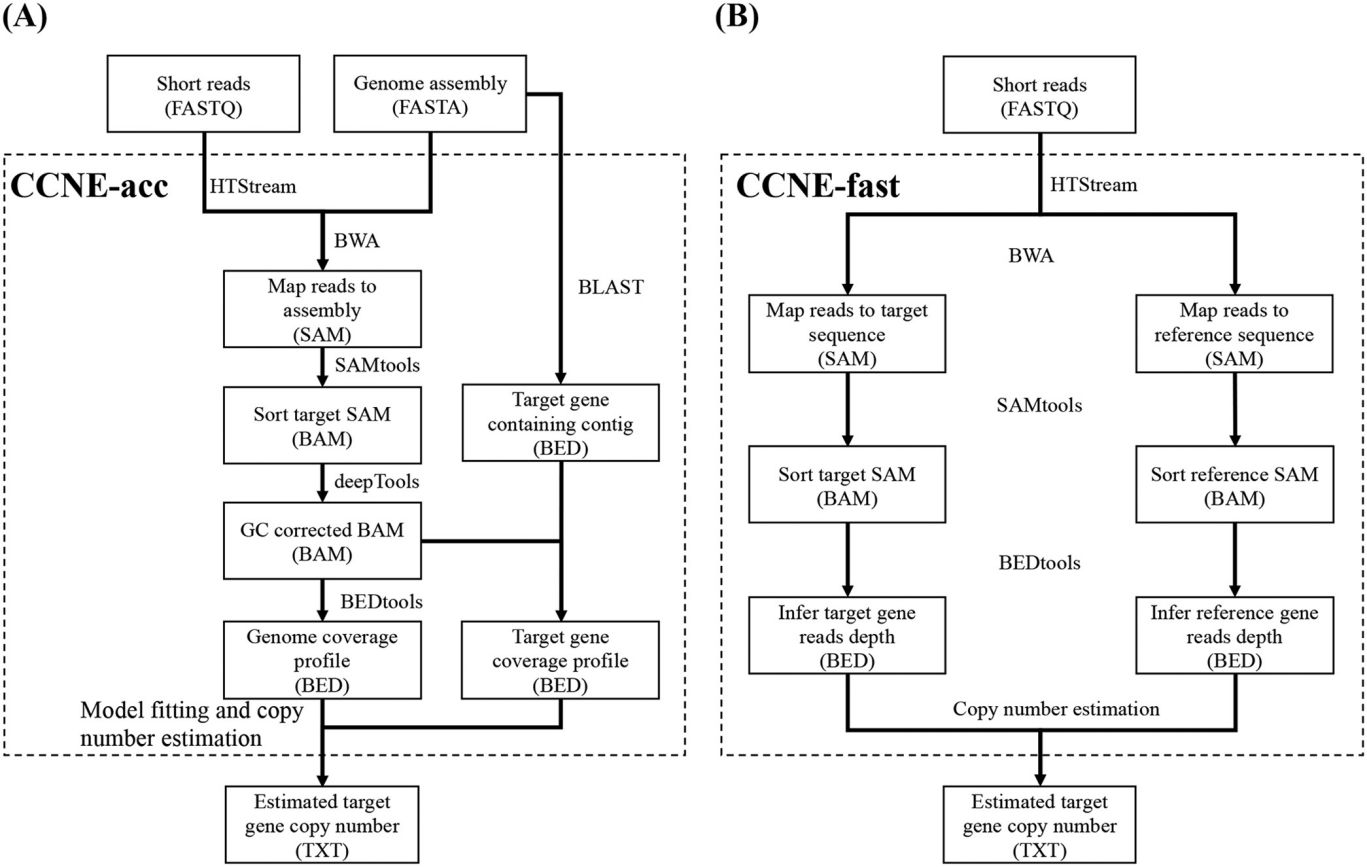
The workflow of CCNE in determining gene copy number using WGS reads. (A) Workflow of CCNE-acc. (B) Workflow of CCNE-fast.

The general procedure for CCNE in *bla*Carb copy number estimation from whole-genome sequencing data is shown in Fig. 1. After mapping the sequence reads to the assembled genome (CCNE-acc) or target gene (CCNE-fast), the mapped reads were analyzed and a genome or gene coverage profile was constructed. The coverage of each position usually follows a Poisson distribution, *P*(*x*|λ) (19), which has one parameter, λ, to be estimated (see Fig. S1 in the supplemental material). CCNE-acc estimates the *bla*Carb copy number by estimating the coverages of the whole genome and the *bla*Carb-containing fragment, respectively. As GC content has a major effect on genome coverage, the GC bias is corrected by the method proposed by Benjamini et al. (20) (see Fig. S2 in the supplemental material). The overall workflow of CCNE-acc is shown in Fig. 2A.

CCNE-fast was developed to rapidly and directly estimate the *bla*Carb copy number profrom WGS reads without assembly. It applied a read depth-based algorithm and calculated the ratio of the read depth of a *bla*Carb gene to that of a reference gene, such as *rpoB* (beta subunit of RNA polymerase) in K. pneumoniae, *dinB* (DNA polymerase IV) in E. coli, and *acsA* (acetyl coenzyme A synthetase) in P. aeruginosa. The reference genes are single-copy genes on the chromosome. CCNE-fast preprocesses WGS reads by using HTStream (quality filter and deduplication), maps them to *bla*Carb and reference sequences using BWA (21), and sorts the reads according to nucleic acid coordinates by using SAMtools (22). It counts the base pairwise read depth by using BEDtools (23) and estimates the *bla*Carb copy number through a CCNE algorithm (Fig. 2B). CCNE-fast is a highly efficient tool that simply maps the WGS reads to *bla*Carb and reference sequences, and it can complete an analysis of a 100× K. pneumoniae WGS sample within 5 min on 4 central processing units. All the required tools have been combined in the CCNE package, which makes it a ready-to-use tool for beginners with little bioinformatics knowledge.

### CCNE supports analysis of all known *bla*Carbs and other antimicrobial resistance genes.

For the *bla*Carbs, CCNE now supports the analysis of all known *bla*Carbs, including *bla*_KPC_, *bla*_NDM_, *bla*_IMP_, *bla*_OXA_, etc. In addition, 377 other antimicrobial resistance genes, such as *bla*_CTX-M_, *bla*_TEM_, and *vanM*, are also supported (https://github.com/biojiang/ccne) (see Table S3 in the supplemental material). CCNE-acc uses assembled contigs to estimate *bla*Carb copy number, without species limitations. CCNE-fast uses a reference gene to estimate *bla*Carb copy number and now supports the analysis of 111 species (see Table S4), including all commonly isolated species published by the China Antimicrobial Surveillance Network (CHINET; http://www.chinets.com/Data/AntibioticDrugFast), such as E. coli, K. pneumoniae, Staphylococcus aureus, Acinetobacter baumannii, and P. aeruginosa. The species list will be updated and extended to cover additional antimicrobial-resistant organisms in the future.

### CCNE achieved high accuracy in simulation compared with available algorithms.

**FIG 3:**
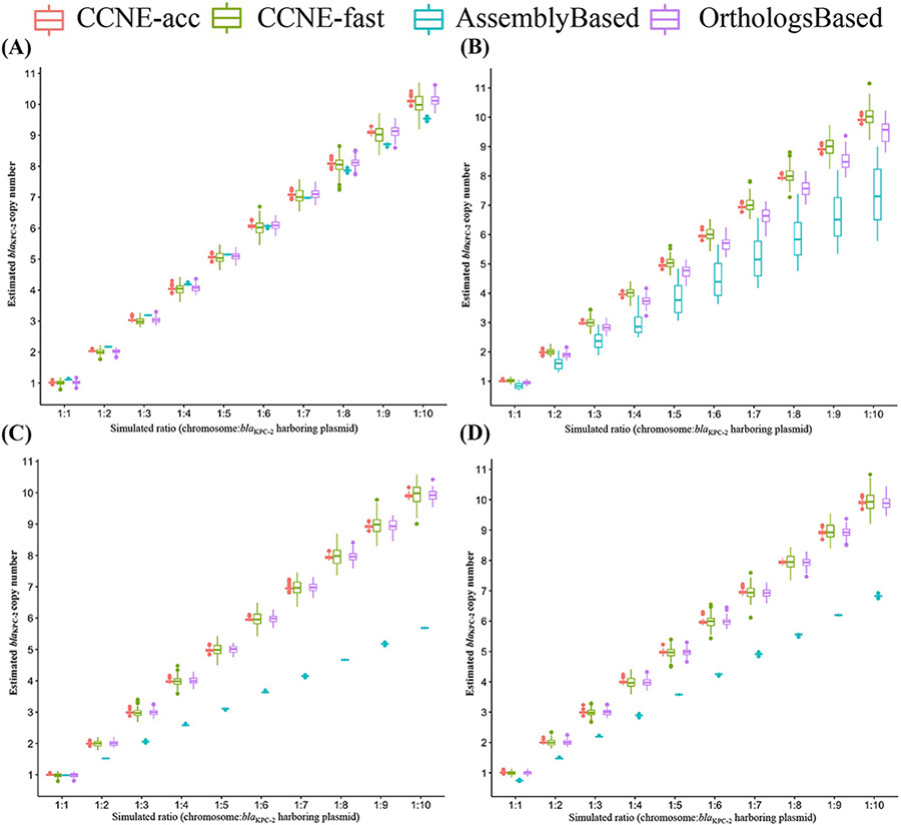
Performance of *bla*Carb copy number estimation methods in simulation. (A) Performance of all promethods in data set without noise. (B) Performance of all methods in data set with noise. (C) Performance proof all methods in data set with various numbers of *bla*_KPC_ in tandem repeat. (D) Performance of all methods in data set with various numbers of *bla*_KPC_ in tandem and multiple plasmids.

To evaluate the performance of CCNE, simulation reads were utilized to compare CCNE with available algorithms, including AssemblyBased (16) and OrthologsBased (18) methods. The pseudogenomes with different numbers of copies of *bla*_KPC_ (from 1 to 10) were obtained by mixing simulated reads from sequences of chromosome and *bla*_KPC-2_-carrying plasmid in different ratios (1:1 to 1:10). In non-noise data sets, all methods acquired compatible high accuracies (>96%) and a low root mean squared error (RMSE; <0.22) (Fig. 3A). In noise data sets, CCNE-acc achieved the best accuracy (100%) and the lowest RMSE (0.07), compared to the AssemblyBased (23.4% accuracy and 1.697 RMSE) and the OrthologsBased (78.9% accuracy and 0.395 RMSE) methods (Fig. 3B). CCNE-fast also achieved a high accuracy (97.1% accuracy and 0.21 RMSE) in noise data sets. In tandem repeat (Fig. 3C) and mixed (Fig. 3D) data sets, CCNE-acc, CCNE-fast, and OrthologsBased methods acquired high accuracies and low RMSEs, while the AssemblyBased method largely underestimated gene copy numbers. Detailed results are shown in Table S5 in the supplemental material. Simulation results suggested that CCNE-acc and CCNE-fast are more appropriate for real-world sequencing data, which contain mixed or repeated reads from chromosome and mobile genetic elements with different sequencing lengths and depths.

### CCNE revealed higher consistency with qPCR than other algorithms.

**FIG 4:**
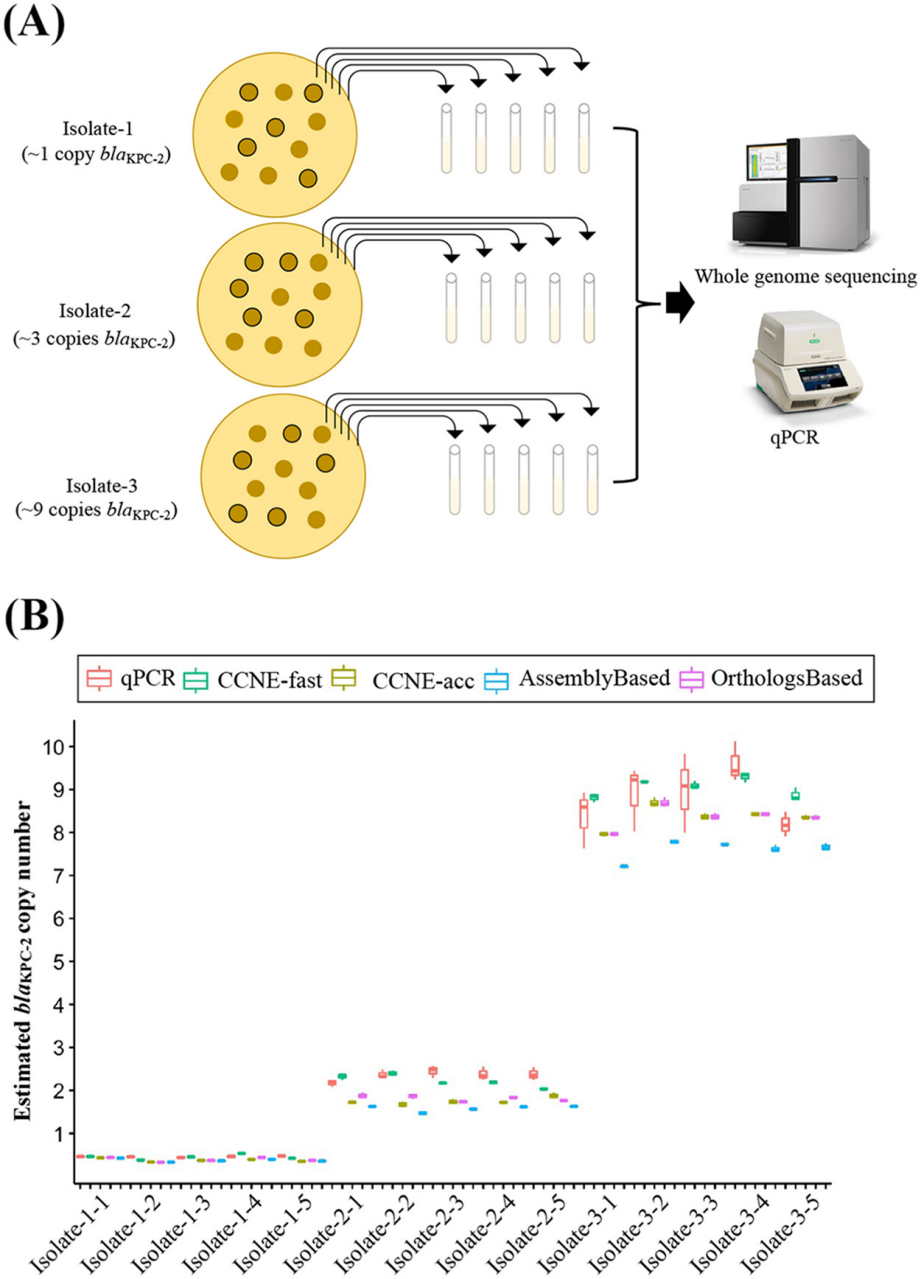
Evaluation of performance of CCNE, AssemblyBased method, and OrthologsBased method on real data. (A) Procedure of the evaluation. (B) The *bla*_KPC_ copy numbers detected by qPCR and estimated by CCNE, AssemblyBased, and OrthologsBased methods.

To confirm the reliability of *bla*Carb copy number estimation from whole-genome sequencing data, qPCR was employed to determine the *bla*Carb copy numbers. Three isolates with approximately 1, 3, and 9 copies of *bla*_KPC_ (based on CCNE) were selected from 357 CRACKLE-2 China isolates (Fig. 4A). The *bla*_KPC_ copy numbers determined by qPCR were 0.46 ± 0.02, 2.35 ± 0.15, and 8.81 ± 0.76 (means ± standard errors), while copy numbers estimated with CCNE-fast were 0.45 ± 0.05, 2.22 ± 0.13, and 9.05 ± 0.21, respectively (Fig. 4B). The *bla*_KPC_ copy numbers were 0.38 ± 0.03, 1.75 ± 0.08, and 8.36 ± 0.05 when determined by CCNE-acc, 0.37 ± 0.04, 1.58 ± 0.07, and 7.60 ± 0.22 when estimated with the AssemblyBased method, 0.39 ± 0.05, 1.82 ± 0.07, and 8.36 ± 0.25 when estimated with the OrthologsBased method. The consistency between the results from qPCR and whole-genome sequencing-based methods demonstrated that *bla*Carb copy number estimations from whole-genome sequencing are reliable.

### *bla*_KPC_ copy numbers estimated by CCNE positively correlated with *bla*_KPC_ gene expression levels and antimicrobial resistance levels.

**FIG 5:**
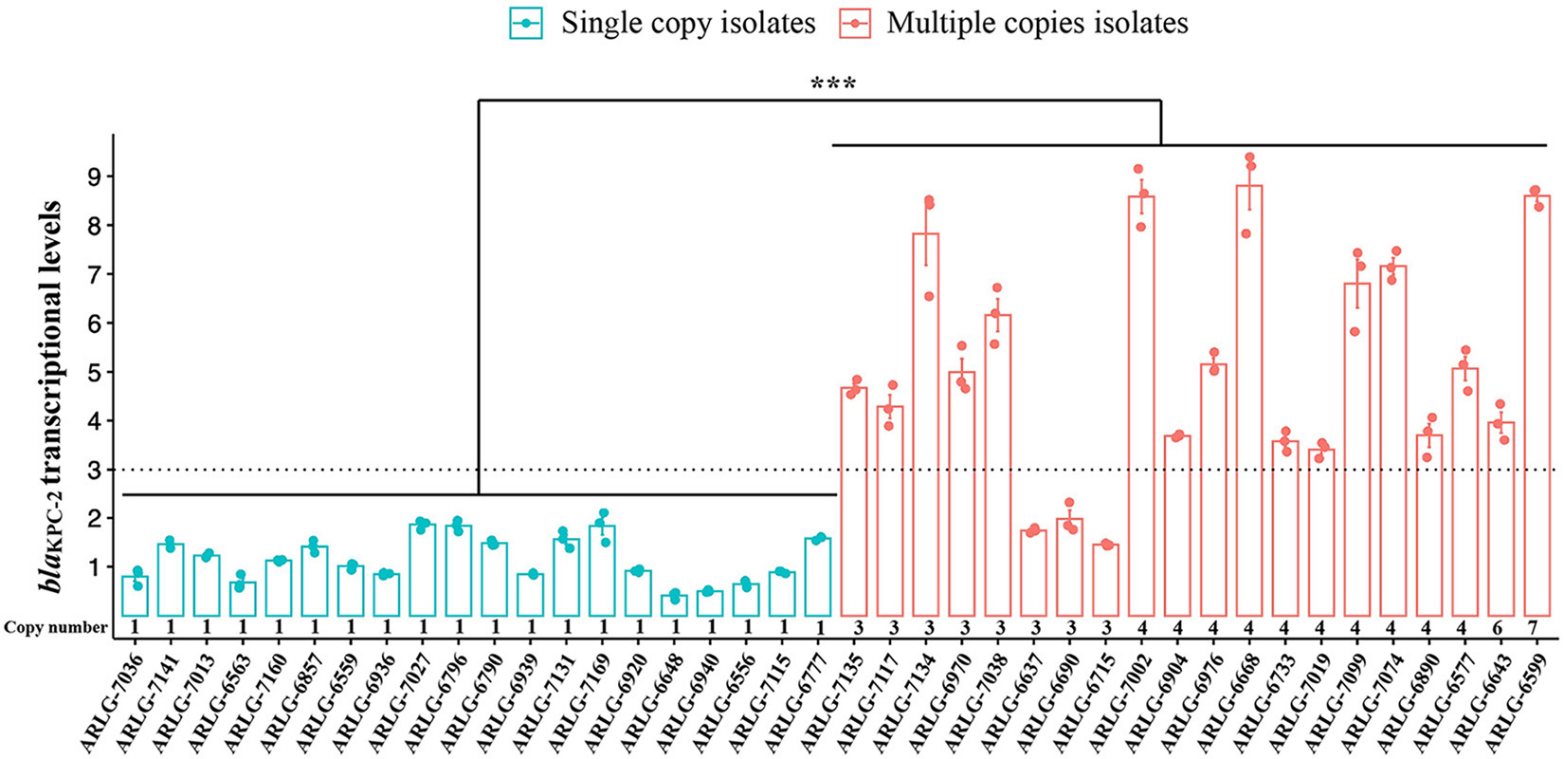
Transcriptional levels of *bla*_KPC-2_ in isolates with a single copy and those with multiple copies.

To determine the association between CCNE estimates of *bla*_KPC_ copy number and gene expression, the transcriptional level of *bla*_KPC_ in 20 isolates with a CCNE-estimated single *bla*_KPC_ copy and 20 isolates with CCNE-estimated multiple *bla*_KPC_ copies (3 to 7 copies) was determined by qRT-PCR. The MIC ranges (and MIC_50_, in parentheses) of meropenem, imipenem, and CAZ-AVI for the 20 CRKP isolates with low numbers of copies of *bla*_KPC_ were 32 to 128 μg/mL (128 μg/mL), 16 to 512 μg/mL (256 μg/mL), and 1 to 4 μg/mL (2 μg/mL), respectively. For the 20 CRKP isolates with high numbers of copies of *bla*_KPC_, these values were 64 to 512 μg/mL (256 μg/mL), 256 to 1,024 μg/mL (512 μg/mL), and 2 to 8 μg/mL (4 μg/mL), respectively. The transcriptional levels of *bla*_KPC_ in isolates estimated by CCNE to carry a single *bla*_KPC_ copy were significantly lower than levels in isolates estimated by CCNE to carry multiple *bla*_KPC_ copies (1.15 ± 0.46 versus 5.08 ± 2.28; *P* < 0.001) (Fig. 5). Estimated *bla*_KPC_ copy numbers were positively correlated with *bla*_KPC_ expression levels (Pearson coefficient, 0.76; 95% confidence interval [CI], 0.68 to 0.83; *P* < 0.001).

**FIG 6:**
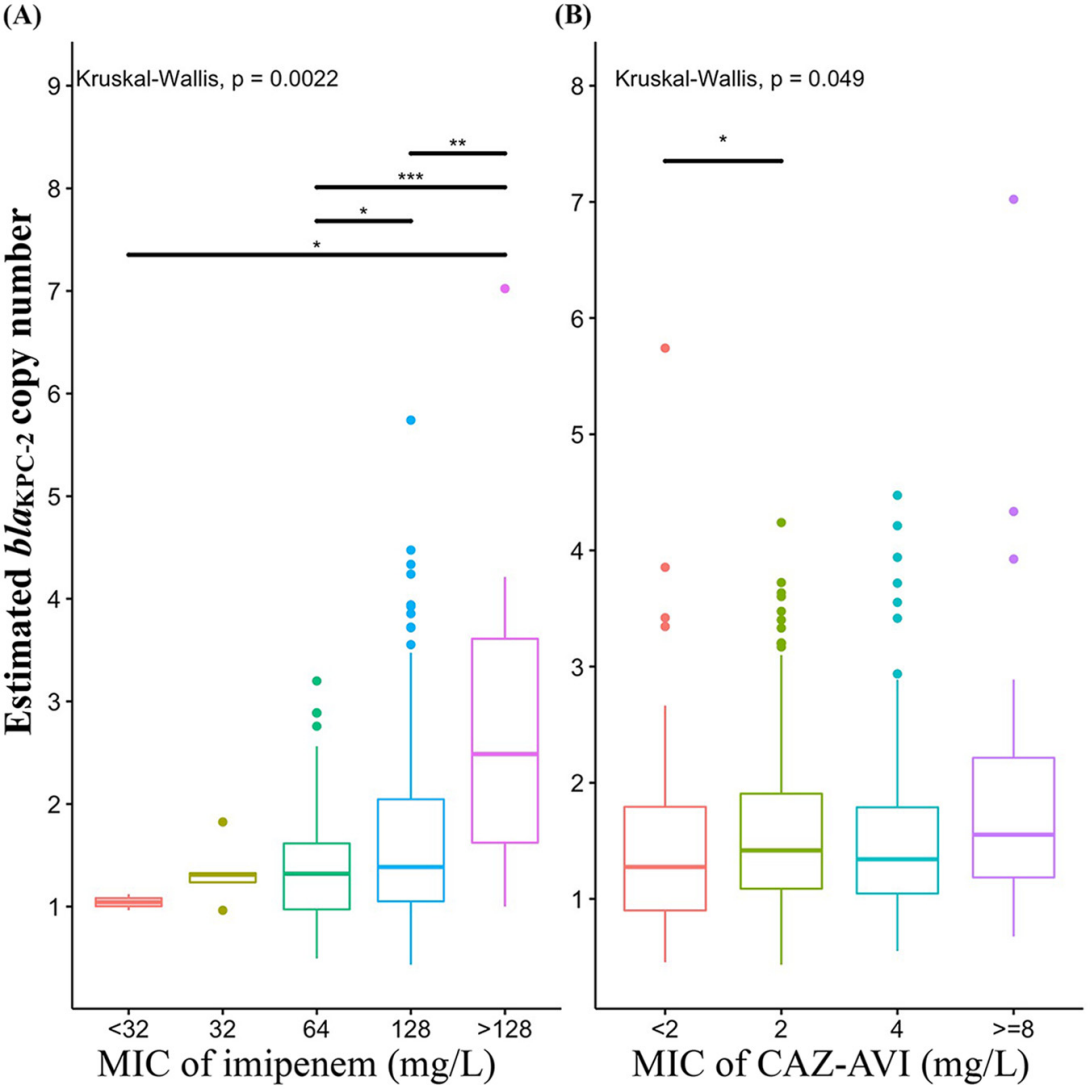
Correlation between estimated *bla*_KPC-2_ copy numbers and MICs of imipenem and CAZ-AVI in 357 *bla*_KPC-2_-harboring ST11 K. pneumoniae isolates. (A) Distribution of estimated *bla*_KPC-2_ copy numbers in imipenem groups with different MICs. (B) Distribution of estimated *bla*_KPC-2_ copy numbers in CAZ-AVI groups with different MICs.

To study the association between the *bla*_KPC_ copy numbers estimated by CCNE and antimicrobial resistance, 357 sequenced ST11 *bla*_KPC-2_-harboring K. pneumoniae isolates with same *ompK35* and *ompK36* genotypes were selected. The *bla*_KPC_ copy numbers estimated by CCNE-acc showed that 42.23% of the isolates harbored more than one copy of *bla*_KPC_, and the average copy number was 1.6 (range, 0.4 to 7) (see Fig. S4 in the supplemental material), indicating that isolates harboring multiple copies of *bla*_KPC_ are common in clinical *bla*_KPC-2_-harboring ST11 K. pneumoniae. Then, the association between the copy numbers and the imipenem and ceftazidime-avibactam MICs were analyzed. The Kruskal-Wallis test showed that CCNE-estimated copy numbers were significantly associated (*P* < 0.05) with MICs of imipenem (Fig. 6A) and CAZ-AVI (Fig. 6B). The estimated *bla*_KPC-2_ copy numbers were positively correlated with the imipenem MIC (Pearson coefficient, 0.278; 95% CI, 0.181 to 0.370; *P* < 0.001) and CAZ-AVI MIC (Pearson coefficient, 0.164; 95% CI, 0.060 to 0.264; *P* < 0.001). A recent study in China found that increased *bla*_KPC-2_ copy number changes were associated with decreased CAZ-AVI susceptibility in carbapenem-resistant P. aeruginosa (18). We applied CCNE-fast to determine the *bla*_KPC-2_ copy numbers and assessed the correlation with the CAZ-AVI MICs using the data from the above study. Similarly, positive associations between *bla*_KPC-2_ copy numbers and CAZ-AVI MIC values were detected, and our CCNE-fast method showed consistent results with their findings (see the supplemental material for more details).

### *bla*_KPC-2_ copy number variations among K. pneumoniae, E. coli, and P. aeruginosa genomes.

To investigate the *bla*_KPC-2_ copy number profiles among different species, publicly available genome assemblies of K. pneumoniae, E. coli, and P. aeruginosa were obtained from the NCBI GenBank database. Genomic analysis showed that 26.56% (2,840/10,692) of K. pneumoniae, 0.54% (127/23,447) of E. coli, and 0.58% (34/5,850) of P. aeruginosa isolates harbored *bla*_KPC-2_ (see Table S6 in the supplemental material). Among them, 808 K. pneumoniae, 46 E. coli, and 11 P. aeruginosa strains had matched SRA short read data available for CCNE analysis. In *bla*_KPC-2_-harboring strains, 51.86% of K. pneumoniae (see Fig. S4A), 76.09% of E. coli (see Fig. S4B), and 54.55% of P. aeruginosa (see Fig. S4C) harbored more than one copy of *bla*_KPC-2_, and the average copy numbers were 2.0, 4.1, and 1.7, respectively, demonstrating that isolates with multiple copies of *bla*_KPC-2_ are widely distributed in these species. The average *bla*_KPC-2_ copy number for E. coli strains appeared to be significantly higher than those for K. pneumoniae and P. aeruginosa (see Fig. S4D), while no significant difference was found between K. pneumoniae strains from NCBI and isolates from CRACKLE-2 China cohort.

Among the 808 *bla*_KPC-2_-harboring K. pneumoniae isolates for which there are raw data in NCBI, 67 of them have been completely sequenced. A total of 65 completed isolates harbor plasmid-borne *bla*_KPC-2_, of which four isolates harbor *bla*_KPC-2_ on 2 plasmids (with 2 to 4 estimated copies), and 2 harbor chromosomal *bla*_KPC-2_ (with 1 estimated copy). By replicon analysis with PlasmidFinder, we found that among the 61 *bla*_KPC-2_ carrying plasmids, 18 plasmids belong to IncFII(pHN7A8)/IncR, 9 belong to IncFIB(pQil)/IncFII_pKP91, 5 belong to IncA/C2, 5 belong to repA_pKPC-2, and the remaining 14 belong to other replicon families. As shown in Fig. S5, the Wilcoxon test revealed that the estimated copy number of *bla*_KPC-2_ in isolates with IncFII(pHN7A8)/IncR plasmids was significantly higher than that of *bla*_KPC-2_ in isolates with IncFIB(pQil)/IncFII_pKP91. No significant difference was observed between other groups. For the 46 *bla*_KPC-2_-harboring E. coli isolates, 20 of them have been analyzed, and all the *bla*_KPC-2_ genes were located on plasmids. Among them, 6 plasmids belonged to IncN, 4 belonged to IncA/C2, and the remaining 10 belonged to other replicon families. For 11 *bla*_KPC-2_-harboring P. aeruginosa isolates, 2 of them have been completed and both the *bla*_KPC-2_ genes were located on untypeable plasmids.

## DISCUSSION

In this study, we developed a robust and ready-to-use toolset, CCNE, including CCNE-acc and CCNE-fast, to estimate *bla*Carb copy numbers from WGS data, and we evaluated its performance on *bla*_KPC_ copy number estimation through simulation, qPCR, and comparison with available algorithms. Our discovery is an important contribution to the field in several ways.

The *bla*Carb copy number plays an important role in the resistance to carbapenems and novel β-lactam and β-lactamase inhibitor combinations in carbapenemase-producing isolates. The routine technique for the detection of *bla*Carb copy number is qPCR. However, the need for optimizing efficient primers and establishing standard curves limits use of qPCR for large-scale studies. Given the increasing application of WGS in antimicrobial studies, WGS-based copy number determination is a more straightforward and scalable approach. Some WGS analysis methods, including AssemblyBased and OrthologsBased methods, have been applied to analyze the *bla*_KPC_ copy numbers in K. pneumoniae, E. coli, and P. aeruginosa (16). However, they all require assembling the WGS reads into contigs and/or predicting the single-copy genes from the contigs, making these analyses time-intensive and computing resource-intensive. More importantly, their accuracies for copy number estimation have not been validated, and no ready-to-use tools are available. The current study overcomes these challenges by estimating the *bla*Carb copy number directly from WGS data, validating the estimation algorithm with simulation and qPCR, and implementing the algorithm into a ready-to-use tool provided to the community.

CCNE was initially developed to analyze the *bla*Carb copy number in carbapenemase-producing isolates, and it supports the analysis of all known *bla*Carbs. Given that gene copy number increases are also commonly found in other antimicrobial resistance genes, for example, the multiple *bla*_TEM-1B_ copy-mediated piperacillin-tazobactam resistance in E. coli (24) and tandem *vanM* repeats that drive vancomycin resistance in Enterococcus faecium (25), CCNE has been extended to support an additional 377 antimicrobial resistance genes, including *bla*_CTX-M_, *bla*_TEM_, and *vanM*. CCNE-fast uses predefined reference genes for common clinical species, such as E. coli, K. pneumoniae, S. aureus, A. baumannii, and P. aeruginosa. CCNE-fast will be updated to support additional antimicrobial-resistant organisms in the future. CCNE-acc uses the assembled contigs to estimate *bla*Carb copy numbers, without species limitations.

In simulations, CCNE achieved the highest accuracy and the lowest RMSE compared with AssemblyBased and OrthologsBased methods. In real data, CCNE determinations of gene numbers were also consistent with qPCR findings. In a validation study, the *bla*_KPC_ copy numbers estimated with CCNE were positively correlated with *bla*_KPC_ gene expression levels and elevated imipenem and ceftazidime-avibactam MICs, which are consistent with the results from previous qPCR-based studies (7, 12, 26). As both CCNE-fast and qPCR use the reads count or *C_T_* values of *bla*_KPC-2_ and *rpoB* to estimate the copy number and the GC bias was not corrected, the results of CCNE-fast showed higher consistency with qPCR results in this study.

Several isolates in this study harbored (estimates of) less than one copy of *bla*_KPC_, which may have been caused by two factors. First of all, the sequencing bias introduced by library preparation can lead to the gene copy number being over- or underestimated (27). Second, as CCNE determines the average gene copy number of a clone community or population, it will produce an estimate of less than one gene copy if the bacterial cells in the community lost the gene, especially when the gene is located on a transposon or plasmid (28).

While augmenting the *bla*Carb copy number can lead to carbapenem resistance, enhanced production of other multiple carbapenemases, such as the combination of KPC and NDM, can also result in this phenotype (29). In addition, other mechanisms, including porin mutations (30) and efflux pump overexpression (31), may also contribute to resistance phenotypes.

In conclusion, CCNE is a ready-to-use tool to determine the gene copy number of all known *bla*Carbs and most other antimicrobial resistance genes from 111 species. The evaluation for *bla*_KPC_ copy number estimation in KPC-producing K. pneumoniae by simulation, qPCR, and comparison with available algorithms revealed that CCNE is a robust and useful tool for *bla*Carb copy number estimation using WGS data. CCNE can be further used to interrogate the correlations between genotypes and susceptibility phenotypes and to improve our understanding of antimicrobial resistance mechanisms.

## MATERIALS AND METHODS

### Overview of CCNE.

CCNE has two components, CCNE-acc and CCNE-fast, which are designed for different application scenarios. CCNE-acc estimates *bla*Carb copy numbers by utilizing whole-genome reads mapping coverage information. CCNE-fast is designed for rapid screening of *bla*Carb copy numbers when the target gene is known. CCNE-acc assumes that read coverage for each position of the whole genome and the *bla*Carb-containing fragment follow a Poisson distribution, and it estimates the *bla*Carb copy number by fitting two Poisson models. The *bla*Carb copy number in CCNE-acc is estimated according to this equation: copy number(CCNE-acc)*_bla_*_Carb_ = (λ*_bla_*_Carb_)/(λ_Genome_), where λ*_bla_*_Carb_ and λ_Genome_ are the parameters of two Poisson distributions and are approximated by the medians of read coverages of *bla*Carb-containing fragments and the whole genome.

CCNE-fast is a reference-based method. It only counts the reads mapped to the target *bla*Carb and the single-copy reference gene. The *bla*Carb copy number in CCNE-fast is estimated according to this equation:

$$\mathrm{Copy}\;\mathrm{number}{\left(\mathrm{CCNE}-\mathrm{fast}\right)}_{bla\text{Carb}}=\;\frac{\frac{1}{L_{CEG}\;-\;2f}\sum_{i=f+1}^{L_{bla\text{Carb}}-f}c_{i}}{\frac{1}{L_{ref}\;-\;2f}\sum_{i=f+1}^{L_{ref}-f}c_{i}}$$

where *L_bla_*_Carb_ is the *bla*Carb gene length, *L*_ref_ is the reference gene length, *f* is the length of flanking sequence excluded in the calculation, and *c_i_* is the read depth of the *i*th base in the gene. Here, *f* is applied for controlling the bias introduced by the reads mapped to the gene boundaries.

### Simulation of genomes with different copies of *bla*_KPC_.

To evaluate the performance of CCNE, we used the *bla*_KPC_ gene as an example of *bla*Carb. bbmap (https://sourceforge.net/projects/bbmap/) was used to generate the simulation reads based on the complete genome sequence of K. pneumoniae clinical isolate HS01777, which contains one *bla*_KPC-2_-carrying plasmid and 4 other plasmids. The non-noise data sets contain the chromosome and *bla*_KPC-2_-carrying plasmid reads, mixed from 1:1 to 1:10 ratio (chromosome:*bla*_KPC-2_-carrying plasmid ratio), with an average chromosome read coverage of 100. The noise data sets were generated by adding all 4 other plasmids for simulated reads from HS01777 to non-noise data sets on a random coverage (from 100, 200, or 300 to 1,100). The tandem repeat data sets were generated by repeating the *bla*_KPC-2_ regional sequences 1 to 10 times in tandem. The mix data sets contain the chromosome and *bla*_KPC-2_-carrying plasmid (with two copies of *bla*_KPC-2_ in tandem) sequences, which were mixed in ratios from 1:1 to 1:5. All the data sets in each ratio group were generated 100 times.

### Comparison of CCNE with other *bla*_KPC_ copy number estimation methods.

The AssemblyBased method was implemented according to the methods of Stoesser et al. (16). In brief, the raw sequencing reads of HS01777 were assembled into contigs by SPAdes v3.11.1 (32), and the *bla*_KPC_-carrying contig was identified by BLAST using the *bla*_KPC-2_ sequence as the query sequence. The *bla*_KPC_ copy number was estimated by dividing the read depth of the contig containing *bla*_KPC_ by the average read depth for the entire genome assembly (weighted by contig length). The OrthologsBased method was implemented according to the methods of Zhu et al. (18). The *bla*_KPC_ copy number in the orthologs-based method was estimated by calculating the ratio of read depth of *bla*_KPC_ to the average depth of single-copy genes. The single-copy genes in HS01777 were identified by using BUSCO with the single-copy genes derived from the BUSCO v.4.0.0 *Enterobacterales* ortholog database, 10th edition (33). The scripts and results have been placed on https://github.com/biojiang/ccne_simulation. Boxplots were generated in R, and the accuracies were calculated based on the read depth ratios, according to the following equation: accuracy = (true positives)/(true positives + false negatives), and the true positives value was calculated by comparing the rounded number of read depth ratios with the true copy numbers.

The root mean squared error (RMSE) was calculated according to the following equation:

$$\mathrm{RMSE}=\sqrt{\frac{1}{N}\overset{N}{\underset{k=1}{\sum }}{\left(C_{k}-\hat{C_{k}}\right)}^{2}}$$

where *N* is the sample size, *k* is the sample index, *C_k_* is the simulated copy number of *bla*_KPC_, and *Ĉ*_*k*_ is the estimated *bla*_KPC_ copy number.

### Whole-genome sequencing data of 357 KPC-producing K. pneumoniae isolates.

Whole-genome sequencing data of 357 ST11 KPC-producing K. pneumoniae isolates were acquired from the Second Consortium on Resistance Against Carbapenems in Klebsiella and other *Enterobacterales* (CRACKLE-2) project of the MDRO Network China cohort (34). All isolates harbored the same *ompK35* (ΔOmpK35, OmpK35 truncation) and *ompK36* (OmpK36GD, OmpK36 134-135 GD insertion) mutant genotypes, to avoid effects of the porin mutation on the resistance phenotypes (see Table S1 in the supplemental material) (30).

### Determining *bla*_KPC_ copy numbers by qPCR.

The *bla*_KPC-2_ copy numbers were measured relative to *rpoB* by using qPCR as previously described (7). The *bla*_KPC-2_ and *rpoB* genes were cloned into pMD-18T to generate pMD-18T-*bla*KPC-2 and pMD-18T-*rpoB*, respectively. Plasmids pMD-18T-*bla*KPC-2 and pMD-18T-*rpoB* were used as the templates, and primers KPC-qRT-F/KPC-qRT-R and RPOB-qRT-F/RPOB-qRT-R were used to establish the corresponding standard curves for the gene copy number determinations. Three isolates (ARLG-7036, ARLG-7038, and ARLG-6599) with 1, 3, and 9 copies of *bla*_KPC_ (based on CCNE estimation) were selected from 357 CRACKLE-2 China isolates for qPCR testing. The isolates were grown on Luria-Bertani agar, and 5 single colonies of each isolate (15 samples in total) were randomly selected for culture and DNA extraction. The total 15 DNA samples were tested for *bla*_KPC_ copy number by qPCR. All amplifications were carried out in triplicate using three different DNA preparations. Primers are shown in Table S2 of the supplemental material. The 15 DNA samples used in qPCR detection were also subject to whole-genome sequencing. The sequencing was performed on the Illumina NovaSeq 6000 platform (Illumina, San Diego, CA) with 150-bp paired-end reads, as previously described (34).

### Transcriptional analysis of *bla*_KPC_.

Quantitative reverse transcription-PCR (RT-qPCR) was employed to assess the transcriptional expression levels of *bla*_KPC_ as previously described (35). The K. pneumoniae clinical isolate HS02244 with a single copy of *bla*_KPC-2_ was used as a reference for every batch of the qRT-PCR analysis. In this study, 20 K. pneumoniae clinical isolates with a CCNE-acc-estimated single copy of *bla*_KPC_ and 20 isolates with multiple copies from among the 357 CRACKLE-2 China isolates were randomly selected for *bla*_KPC_ transcriptional analysis. All amplifications were carried out in triplicate from three different RNA preparations. Primers are shown in Table S2 of the supplemental material.

### Association between CCNE-determined *bla*_KPC_ copy numbers and MICs.

MICs of imipenem and CAZ-AVI were determined using the CLSI reference broth microdilution method for K. pneumoniae clinical strains (36). The boxplots of the CCNE-acc-determined *bla*_KPC_ copy numbers in each of the imipenem and CAZ-AVI groups with different MICs were generated by using R 4.0.0. The overall *P* values were calculated by using the Kruskal-Wallis test. The correlation coefficients, *P* values, and confidence intervals were calculated by using the Pearson correlation test. All tests were performed in R.

### *bla*_KPC-2_ copy number detection in the NCBI database using CCNE.

The genome assemblies of 10,692 K. pneumoniae, 23,447 E. coli, and 5,850 P. aeruginosa isolates were downloaded from NCBI GenBank (accessed June 2021) and screened for *bla*_KPC-2_ by using BLASTN. The biosamples harboring *bla*_KPC-2_ were searched against the NCBI SRA database to query and download SRA data, followed by conversion to fastq format using SRA tools. The copy numbers of *bla*_KPC-2_ from public data were determined by using CCNE-fast.

### Data availability.

The data have been deposited with links to BioProject accession number PRJNA658369 in the NCBI BioProject database.
