# Supplementary material for: Carbapenemase-Encoding Gene Copy Number Estimator (CCNE): a Tool for Carbapenemase Gene Copy Number Estimation
Source: Microbiol Spectr. 2022 Jul 5;10(4):e01000-22. doi: 10.1128/spectrum.01000-22 (PMC9431437; doi:10.1128/spectrum.01000-22)
Supplement: Supplemental file 1 — Supplemental material. Download spectrum.01000-22-s0001.pdf, PDF file, 0.9 MB [file spectrum.01000-22-s0001.pdf]

Supplimentary text

The raw reads data of 151 *P. aeruginosa* were downloaded from the Bioproject PRJNA672835. The copy number of bla_{KPC-2} in each isolate was estimated by ccne-fast. Further, the association between the copy numbers and the MICs of Ceftazidime-Avibactam was analyzed. Kruskal-Wallis test showed that the CCNE estimated copy numbers were significantly associated with Ceftazidime-Avibactam (Figure S6) MICs ($P<0.001$). The Pearson correlation test showed that the estimated copy numbers are positively correlated with the MICs of Ceftazidime-Avibactam (Spearman rank correlation [ρ] = 0.491; $P < 0.0001$), which were consistent with their results.

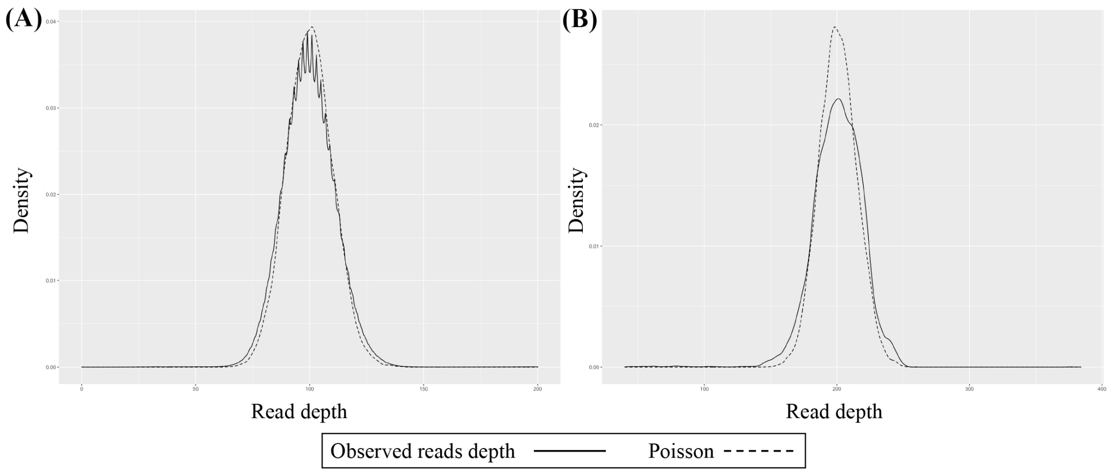

Figure S1. The distribution of reads coverage profiles. (A) The distribution of reads coverage profile of whole genome. (B) The distribution of reads coverage profile of *bla*KPC-2 containing contig. Solid line represents the observed distribution. Dash line represent the theoretical Poisson distribution.

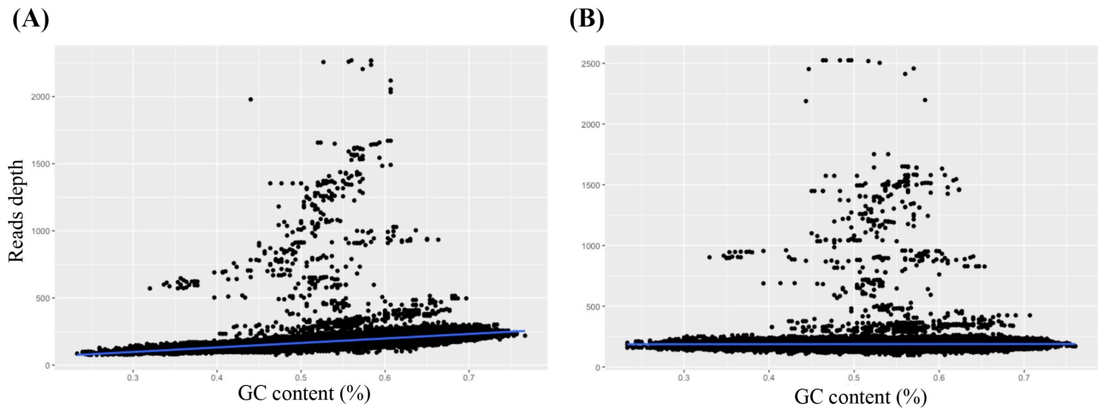

Figure S2. The GC bias in whole genome sequencing. (A) The distribution of reads depth against GC content before GC correction. (B) The distribution of reads depth against GC content after GC correction.

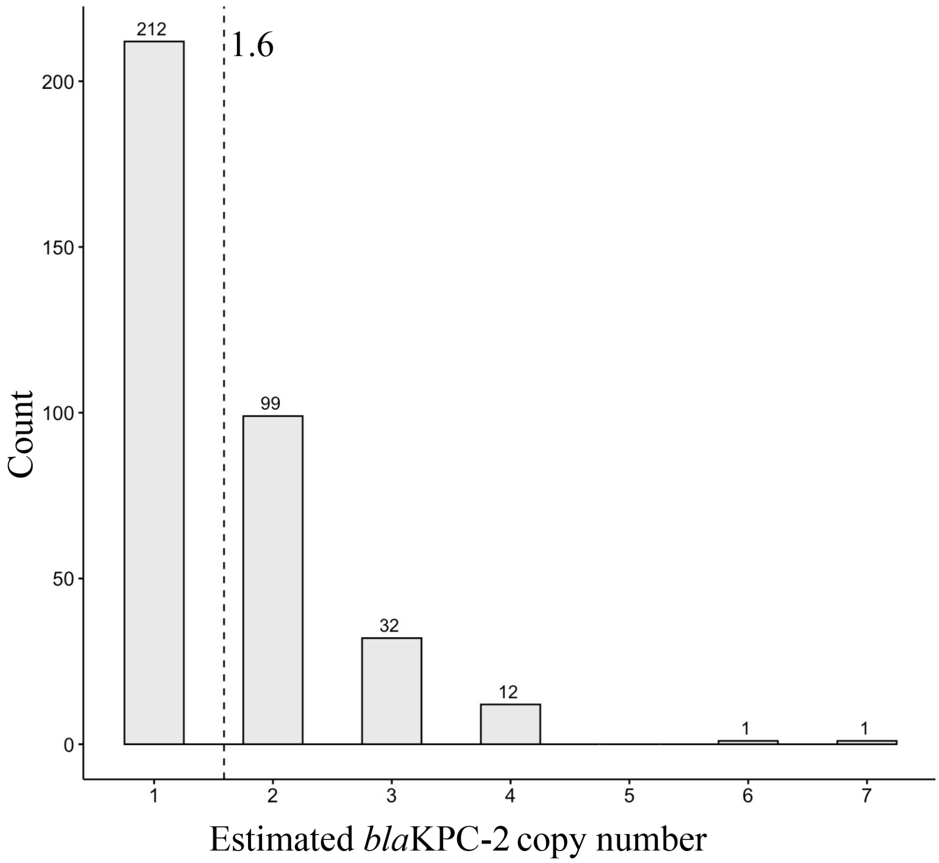

Figure S3. The distribution of *bla*KPC-2 copy number in 357 *bla*KPC-2 harboring ST11 *K. pneumoniae* in our study. The vertical dash line indicates the mean copy number.

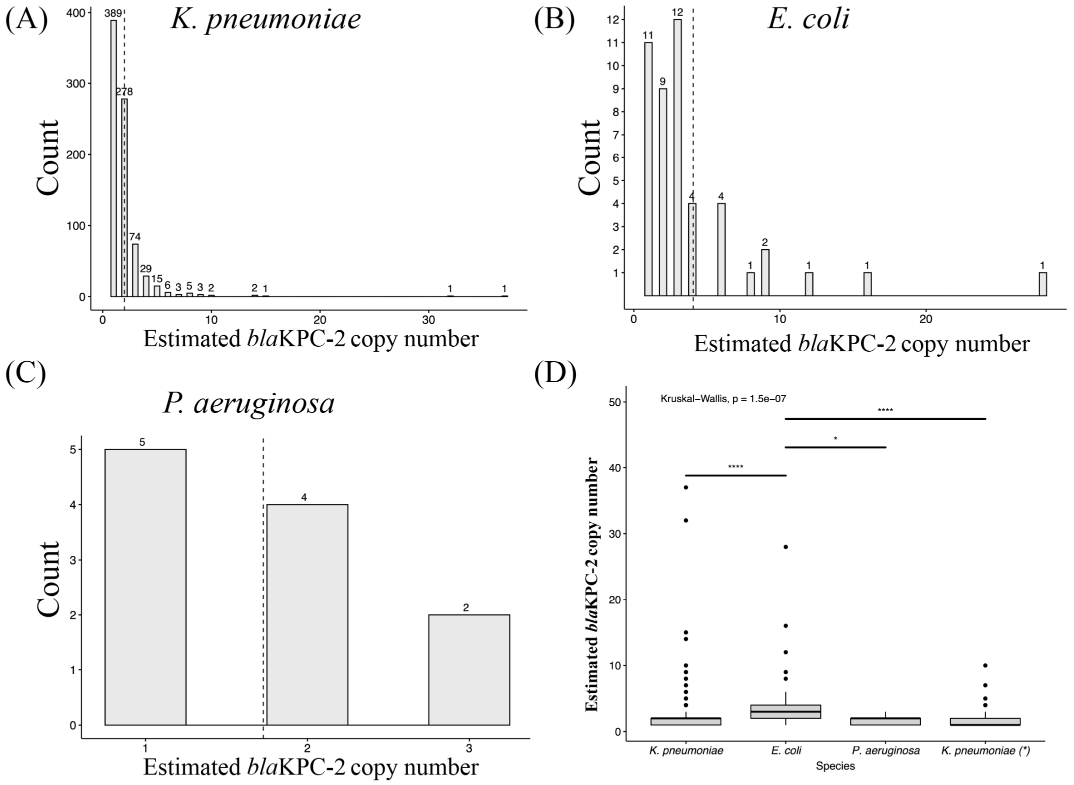

Figure S4. The distribution of *bla*KPC-2 copy number in public WGS data. (A) The distribution of *bla*KPC-2 copy number in 808 *bla*KPC-2 harboring *K. pneumoniae* from NCBI. (B) The distribution of *bla*KPC-2 copy number in 46 *bla*KPC-2 harboring *E. coli* from NCBI. (C) The distribution of *bla*KPC-2 copy number in 11 *bla*KPC-2 harboring *P. aeruginosa* from NCBI. (D) The comparison of *bla*KPC-2 copy number among *K. pneumoniae*, *E. coli* and *P. aeruginosa*. The vertical dash line indicates the mean copy number. *357 $bla_{KPC\text{-}2}$ harboring ST11 *K. pneumoniae* from CRACKLE China cohort.

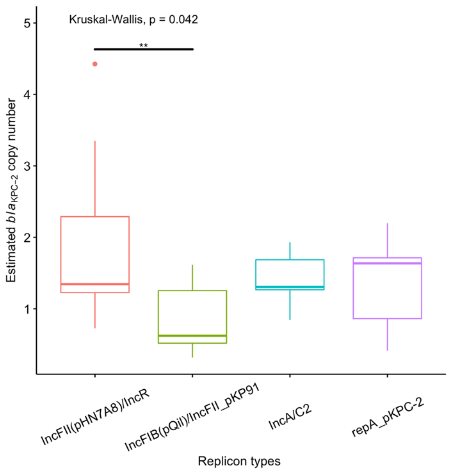

Figure S5. The comparison of *bla*$_{KPC-2}$ copy number between difference plasmid replicons in 61 completed *K. pneumoniae*.

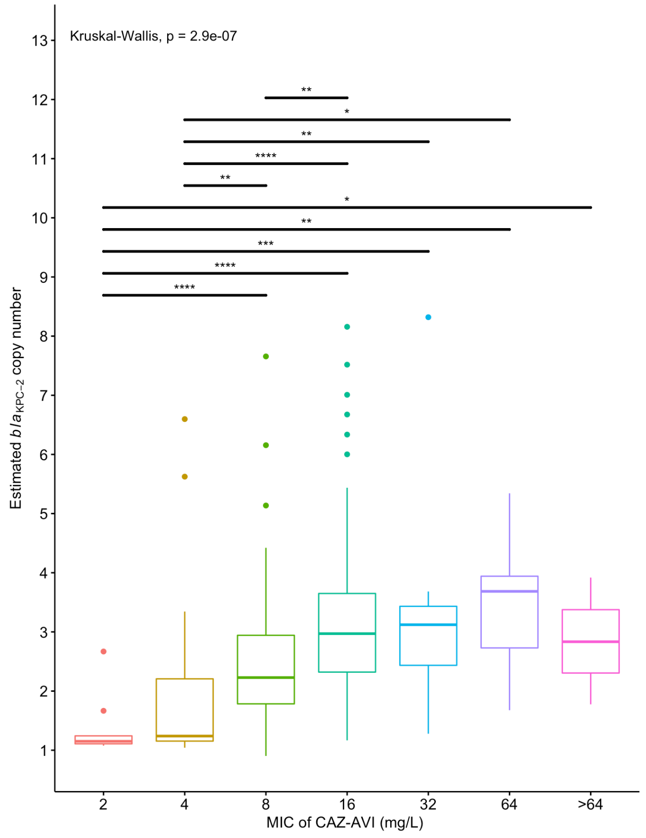

Figure S6. The correlation between estimated *bla*KPC-2 copy number and MICs of Ceftazidime-Avibactam in 151 *bla*KPC-2 harboring *P. aeruginosa* from Zhu's study.

Table S1. CRKP isolates used in this study.

No	ARLG-ID	*bla* KPC-2 copy number
1	ARLG-6706	2.761904762
2	ARLG-6709	1.780898876
3	ARLG-6713	1.54494382
4	ARLG-6715	3.401197605
5	ARLG-6755	1.555555556
6	ARLG-6786	1.554347826
7	ARLG-6790	0.885245902
8	ARLG-6800	1.049723757
9	ARLG-6689	3.602339181
10	ARLG-6690	3.331521739
11	ARLG-6691	3.717791411
12	ARLG-6692	3.344444444
13	ARLG-6694	2.402173913
14	ARLG-6697	2.886486486
15	ARLG-6717	2.513812155
16	ARLG-6752	1.513661202
17	ARLG-6769	1.317204301
18	ARLG-6770	1.770949721
19	ARLG-6775	1.383783784
20	ARLG-6564	3.198895028
21	ARLG-6566	1.898876404
22	ARLG-6569	1.17679558
23	ARLG-6572	1.954285714
24	ARLG-6574	2.242937853
25	ARLG-6579	2.269461078
26	ARLG-6580	3.203592814
27	ARLG-6581	2.717948718
28	ARLG-6587	2.666666667
29	ARLG-6588	2.654761905
30	ARLG-6589	2.535519126
31	ARLG-6590	3.722891566
32	ARLG-6591	3.475308642
33	ARLG-6592	2.746987952
34	ARLG-6594	2.593023256
35	ARLG-6600	2.539877301
36	ARLG-6601	2.909090909
37	ARLG-6602	2.209302326
38	ARLG-6603	2.210191083
39	ARLG-6605	2.141242938
40	ARLG-6606	2.045977011
41	ARLG-6609	2.402439024
42	ARLG-6610	3.098837209
43	ARLG-6612	2.664772727
44	ARLG-6614	2.606060606
45	ARLG-6615	2.463576159
46	ARLG-6617	3.419753086
47	ARLG-6621	2.471264368
48	ARLG-6643	5.740963855
49	ARLG-6644	2.459770115
50	ARLG-6646	2.012269939
51	ARLG-6647	2.04494382
52	ARLG-6648	0.963350785
53	ARLG-6650	1.477777778

54	ARLG-6652	1.331606218
55	ARLG-6653	1.119565217
56	ARLG-6654	1.908571429
57	ARLG-6662	1.548022599
58	ARLG-6663	1.220994475
59	ARLG-6622	2.186813187
60	ARLG-6623	1.469273743
61	ARLG-6628	3.854545455
62	ARLG-6630	2.563218391
63	ARLG-6636	1.225274725
64	ARLG-6637	2.333333333
65	ARLG-6638	1.888888889
66	ARLG-6639	1.644808743
67	ARLG-6669	1.857923497
68	ARLG-6563	0.822580645
69	ARLG-6596	0.861878453
70	ARLG-6814	1.027322404
71	ARLG-6816	1.162162162
72	ARLG-6817	1.107526882
73	ARLG-6818	1.342541436
74	ARLG-6819	1.824468085
75	ARLG-6820	1.356382979
76	ARLG-6824	2.221590909
77	ARLG-6825	0.902702703
78	ARLG-6837	1.868571429
79	ARLG-6828	1.64
80	ARLG-6829	1.010869565
81	ARLG-6830	1.606557377
82	ARLG-6831	1.497175141
83	ARLG-6834	2.011428571
84	ARLG-6577	4.212643678
85	ARLG-6843	1.230769231
86	ARLG-6661	1.594285714
87	ARLG-6768	0.934065934
88	ARLG-6771	0.93442623
89	ARLG-6780	1.429347826
90	ARLG-6782	2.251428571
91	ARLG-6777	0.945355191
92	ARLG-6787	0.788043478
93	ARLG-6839	0.788043478
94	ARLG-6788	0.755434783
95	ARLG-6772	0.555555556
96	ARLG-6796	0.71978022
97	ARLG-6798	0.933701657
98	ARLG-6841	0.968085106
99	ARLG-6832	1.702857143
100	ARLG-6776	1.465909091
101	ARLG-6674	2.394285714
102	ARLG-6677	1.309392265
103	ARLG-6683	1.219653179
104	ARLG-6685	0.789189189
105	ARLG-6701	1.75
106	ARLG-6714	0.64021164
107	ARLG-6716	1.486486486
108	ARLG-6721	0.627659574
109	ARLG-6726	1.026737968

110	ARLG-6732	1.172972973
111	ARLG-6733	3.925531915
112	ARLG-6734	1.297297297
113	ARLG-6738	1.397905759
114	ARLG-6737	1.740540541
115	ARLG-6741	1.653846154
116	ARLG-6731	1.384615385
117	ARLG-6745	1.163934426
118	ARLG-6746	1.936507937
119	ARLG-6748	1.08839779
120	ARLG-6618	1.413043478
121	ARLG-6625	0.940540541
122	ARLG-6627	0.516483516
123	ARLG-6635	0.552083333
124	ARLG-6599	7.02259887
125	ARLG-6640	1.320855615
126	ARLG-6649	0.526315789
127	ARLG-6847	2.195402299
128	ARLG-6853	1.662857143
129	ARLG-6854	1.884615385
130	ARLG-6866	2.31097561
131	ARLG-6884	1.283333333
132	ARLG-6885	1.494252874
133	ARLG-6889	1.38150289
134	ARLG-6897	1.394444444
135	ARLG-6899	1.62962963
136	ARLG-6902	1.643274854
137	ARLG-6904	3.633879781
138	ARLG-6905	1.516483516
139	ARLG-6906	1.447058824
140	ARLG-6908	1.260638298
141	ARLG-6914	1.335195531
142	ARLG-6921	1.388888889
143	ARLG-6923	1.113989637
144	ARLG-6924	1.610810811
145	ARLG-6925	1.704301075
146	ARLG-6936	0.90052356
147	ARLG-6939	0.880829016
148	ARLG-6940	1.020833333
149	ARLG-6942	1.137566138
150	ARLG-6944	1.031413613
151	ARLG-6947	0.96373057
152	ARLG-6556	1.020942408
153	ARLG-6557	1.607526882
154	ARLG-6558	0.935828877
155	ARLG-6559	0.831521739
156	ARLG-7019	3.940217391
157	ARLG-7020	1.382513661
158	ARLG-7024	1.122994652
159	ARLG-7025	0.626315789
160	ARLG-7026	0.608465608
161	ARLG-7027	0.696808511
162	ARLG-7029	1.861878453
163	ARLG-7032	0.547368421
164	ARLG-7036	0.494791667
165	ARLG-7038	2.216931217

166	ARLG-7039	1.032786885
167	ARLG-7041	0.788359788
168	ARLG-7042	1.636363636
169	ARLG-7043	1.016216216
170	ARLG-7047	0.5
171	ARLG-6913	1.420765027
172	ARLG-6920	1.056410256
173	ARLG-6926	0.563829787
174	ARLG-6929	0.863157895
175	ARLG-6930	1.436842105
176	ARLG-6931	1.557894737
177	ARLG-6935	1.348066298
178	ARLG-7048	1.071823204
179	ARLG-7049	0.792349727
180	ARLG-7050	0.940217391
181	ARLG-7051	0.675675676
182	ARLG-7053	1.420454545
183	ARLG-7055	0.75
184	ARLG-7057	1.247311828
185	ARLG-7058	2.151351351
186	ARLG-7060	0.973684211
187	ARLG-7061	1.062146893
188	ARLG-7062	1.491428571
189	ARLG-7063	2.62962963
190	ARLG-7064	0.819148936
191	ARLG-7066	1.648044693
192	ARLG-7067	1.175824176
193	ARLG-7069	0.725274725
194	ARLG-7070	1.366120219
195	ARLG-7071	1.064516129
196	ARLG-7072	1.068571429
197	ARLG-7074	4.335135135
198	ARLG-7080	0.865921788
199	ARLG-7082	0.565217391
200	ARLG-7086	2.862637363
201	ARLG-7089	0.883333333
202	ARLG-7091	1.804469274
203	ARLG-7092	1.584699454
204	ARLG-7093	1.066666667
205	ARLG-7096	1.368715084
206	ARLG-7097	1.740331492
207	ARLG-7099	4.24
208	ARLG-7100	0.530054645
209	ARLG-7102	0.741935484
210	ARLG-7103	0.902173913
211	ARLG-7104	0.572972973
212	ARLG-7106	0.556149733
213	ARLG-7107	1.12849162
214	ARLG-7108	0.524324324
215	ARLG-7110	1.192307692
216	ARLG-6949	1.104395604
217	ARLG-6950	0.918781726
218	ARLG-6951	1.171875
219	ARLG-6954	1.053763441
220	ARLG-6955	1.098445596
221	ARLG-6956	1.497382199

222	ARLG-6957	1.134715026
223	ARLG-6958	1.063157895
224	ARLG-6959	1.025510204
225	ARLG-6960	1.438202247
226	ARLG-6961	1
227	ARLG-6962	0.925925926
228	ARLG-6963	1.233502538
229	ARLG-6964	0.678756477
230	ARLG-6965	1.441025641
231	ARLG-6966	1.558659218
232	ARLG-6967	1
233	ARLG-6969	1.732954545
234	ARLG-6970	2.947916667
235	ARLG-6971	0.43315508
236	ARLG-6974	1.287958115
237	ARLG-6976	3.415300546
238	ARLG-6977	0.454545455
239	ARLG-6991	0.879581152
240	ARLG-6992	0.802197802
241	ARLG-6993	1.355555556
242	ARLG-6979	1.314917127
243	ARLG-6982	1.756906077
244	ARLG-6983	2.342857143
245	ARLG-6984	1.672043011
246	ARLG-6987	1.612359551
247	ARLG-6997	0.988950276
248	ARLG-6998	0.98974359
249	ARLG-6999	1.590673575
250	ARLG-7001	1.542553191
251	ARLG-7002	3.552631579
252	ARLG-7112	1.826086957
253	ARLG-7114	1.338983051
254	ARLG-7115	1.005494505
255	ARLG-7117	2.662921348
256	ARLG-7122	0.972375691
257	ARLG-7124	1.550561798
258	ARLG-7125	1.210810811
259	ARLG-6937	1.087431694
260	ARLG-7126	1.944444444
261	ARLG-7128	1.25136612
262	ARLG-7129	1.155080214
263	ARLG-7130	1.811827957
264	ARLG-7132	1.038461538
265	ARLG-7133	2.758241758
266	ARLG-7134	2.937142857
267	ARLG-7135	2.55801105
268	ARLG-7136	1.666666667
269	ARLG-7138	1.586592179
270	ARLG-7139	2.350877193
271	ARLG-7141	0.632432432
272	ARLG-7142	1.155913978
273	ARLG-7143	1.147540984
274	ARLG-7145	1.945652174
275	ARLG-7147	1.681564246
276	ARLG-7152	1.716763006
277	ARLG-7154	0.839285714

278	ARLG-7156	1.092896175
279	ARLG-7157	1.329608939
280	ARLG-7158	1.163043478
281	ARLG-7159	1.572222222
282	ARLG-7160	0.647058824
283	ARLG-7161	1.054347826
284	ARLG-7162	1.440217391
285	ARLG-7164	1.883333333
286	ARLG-7165	3.168674699
287	ARLG-7166	1.214285714
288	ARLG-7167	0.631016043
289	ARLG-7168	1.302702703
290	ARLG-7169	0.885245902
291	ARLG-7170	1.184210526
292	ARLG-7172	1.248587571
293	ARLG-7003	1.668539326
294	ARLG-7004	1.172222222
295	ARLG-7010	1.306010929
296	ARLG-7013	0.695652174
297	ARLG-7014	1.198895028
298	ARLG-7131	0.884615385
299	ARLG-7137	0.664835165
300	ARLG-7174	1.38547486
301	ARLG-7176	1.296089385
302	ARLG-7177	1.236263736
303	ARLG-7178	1.212290503
304	ARLG-7179	0.853403141
305	ARLG-7181	1.349726776
306	ARLG-7183	1.897727273
307	ARLG-7184	0.652173913
308	ARLG-7186	1.29281768
309	ARLG-6845	1.806629834
310	ARLG-6849	1.461956522
311	ARLG-6850	1.040697674
312	ARLG-6852	1.105263158
313	ARLG-6856	1.325842697
314	ARLG-6894	1.8
315	ARLG-6896	1.535911602
316	ARLG-6911	2.13372093
317	ARLG-7012	1.264044944
318	ARLG-6805	1.414772727
319	ARLG-6576	1.165745856
320	ARLG-6593	2.195402299
321	ARLG-6667	1.870588235
322	ARLG-6668	4.474576271
323	ARLG-6718	1.49132948
324	ARLG-6747	1.261363636
325	ARLG-6758	1.329545455
326	ARLG-6764	1.274285714
327	ARLG-6789	1.369318182
328	ARLG-6797	1.385057471
329	ARLG-6802	1.591954023
330	ARLG-6804	2.888888889
331	ARLG-6807	1.463687151
332	ARLG-6823	1.925287356
333	ARLG-6826	2.139072848

334	ARLG-6835	1.816901408
335	ARLG-6836	2.202702703
336	ARLG-6840	1.958579882
337	ARLG-6851	1.309677419
338	ARLG-6857	0.820224719
339	ARLG-7007	0.8125
340	ARLG-7175	1.022857143
341	ARLG-7148	0.785310734
342	ARLG-7155	1.121387283
343	ARLG-6575	2.372670807
344	ARLG-6996	2.183908046
345	ARLG-7009	1.795321637
346	ARLG-6978	1.225433526
347	ARLG-6932	0.779661017
348	ARLG-6934	0.630434783
349	ARLG-6859	0.666666667
350	ARLG-6865	1.792682927
351	ARLG-6860	0.784090909
352	ARLG-7088	1.226190476
353	ARLG-7030	1.216374269
354	ARLG-6595	1.356725146
355	ARLG-7150	1.354651163
356	ARLG-7151	1.352941176
357	ARLG-7149	1.252873563

Table S2. Primers used in this study.

Primers	Sequence (5'-3')
KPC-qRT-F	GGCCGCCGTGCAATAC
KPC-qRT-R	GCCGCCCAACTCCTTCA
KPC-probe (FAM)	TGATAACGCCGCCGCCAATTTGT
RPOB-qRT-F	CTGATGCCTCAGGATATGATCAAC
RPOB-qRT-R	CTGGCTGGAACCAAAGAACTCT
RPOB-probe (FAM)	CAAGCCGATTTCCGCAGCAGTGA
rrsE_qF	TTGACGTTACCCGCAGAAGAA
rrsE_qR	GCTTGCACCCTCCGTATTACC
pMD-18T-KPC2-F	ATCGCCGTCTAGTTCTGCTG
pMD-18T-KPC2-R	TCGCTGTGCTTGTCATCCTT
pMD-18T-rpoB_F	ATATCGACCACCTCGGCAAC
pMD-18T-rpoB_R	TCATCCAGGTTGGAGTTCGC

Table S3. Antimicrobial resistance genes supported in CCNE.

Antimicrobial agents	Subtypes	Gene or protein names*
Aminoglycosides		AAC(2')(7), AAC(3)(20), AAC(6')(65), ANT(2")(1), ANT(3")(35), ANT(4')(4), ANT(6)(5), ANT(9)(2), APH(2")(7), APH(3")(3), APH(3')(18), APH(4)(2), APH(6)(7), APH(7")(1), APH(9)(3), ApmA(1), ArmA(1), KamB(1), NpmA(1), RmtA(1), RmtB(1), RmtC(1), RmtD(2), RmtE(2), RmtF(1), RmtG(1), RmtH(1), SAT(3), Sgm(1), Sta(1), StrA(1), StrB(1)
β-lactams	β-lactamase	ACC(4), ACT(35), ADC(14), AER(1), AIM(1), AQU(3), ARL(6), AST-1(1), AmpC1(1), BAT-1(1), BCL-1(1), BIL(1), BJP(1), BPU-1(1), BRO(3), BUT-1(1), BcI(1), BcII(1), Bla1(1), Bla2(1), CAM-1(1), CARB(20), CAU(1), CBP(1), CKO(1), CMH(1), CMY(114), CPS(1), CblA(1), CepS(1), CfiA(1), CfxA(4), CphA(10), DHA(19), EBR(1), ESP(1), FEZ(1), FONA(6), FOX(9), FTU(1), GES(4), GIM(2), GOB(13), HERA(7), HMB(1), IND(16), JOHN-1(1), KHM(1), L1(1), LAP(2), LCR(1), LHK(1), LRA(13), MIR(16), MOX(9), MSI(2), MUS(2), NPS(1), OXA(114), PC1(1), PDC(10), PEDO(3), PNGM(1), R39(1), RCP(1), RHO(1), RM3(1), ROB(2), RSA(2), SCO(1), SDA-A(1), SED(1), SFB(1), SIM(1), SLB(1), SMB(1), SPG(1), SRT(1), TEM(51), THIN-B(1), TMB(1), TRU(1), TUS(1), blaA(1), blaB21(1), blaF(1)
	Extended-spectrum β-lactamase	ACI(1), ADC(32), BEL(3), BES(1), CARB(1), CGA(1), CIA(4), CME(1), CMY(5), CTX-M(141), CepA(1), CfxA(3), DES(1), ERP(1), FAR(1), GES(9), OCH(8), OXA(25), OXY(24), PDC(3), PER(7), SFO-1(1), SHV-OKP-LEN(42), SRT(1), TEM(84), TLA(4), VEB(10)
	Carbapenem	BIC(1), BKC(1), CGB(1), CTX-M(1), DIM(1), EBR(1), FIM(1), FPH(1), FRI(3), GES(13), IMI(7), IMP(48), KPC(20), NDM(27), OXA(300), SFH(1), SME(5), SPM(1), TMB(1), VCC(1), VIM(40)
	β-lactamase on chromosome	SHV-OKP-LEN(163)
	Inhibitor-resistant β-lactamase	SHV-OKP-LEN(6), TEM(23)
	Inhibitor-resistant extended-spectrum β-lactamase	TEM(9)
Colistin		ICR-Mo(1), Mcr1(14), Mcr2(2), Mcr3(12), Mcr4(5), Mcr5(2), Mcr6(1), Mcr7(1), Mcr8(1), Mcr9(1)
Fosfomycin		Fom(2), FosA(4), FosB(7), FosC(2), FosD(1), FosK(1), FosX(2)
Fluoroquinolone		CrpP(1), NorA(1), QepA(1), Qnr(1), QnrA(7), QnrB(71), QnrC(1), QnrD(2), QnrE(2), QnrS(12), QnrVC(6)
Glycopeptide		VanA(1), VanB(1), VanC(1), VanD(1), VanE(1), VanF(1), VanG(1), VanHA(1), VanHB(1), VanHD(1), VanHE(1), VanHM(1), VanHO(1), VanI(1), VanJ(1), VanL(1), VanM(1), VanN(1), VanO(1), VanRA(1), VanRB(1), VanRC(1), VanRD(1), VanRE(1), VanRF(1), VanRG(1), VanRI(1), VanRL(1), VanRM(1), VanRN(1), VanRO(1), VanSA(1), VanSB(1), VanSC(1), VanSD(1), VanSE(1), VanSF(1), VanSG(1), VanSL(1), VanSM(1), VanSN(1), VanSO(1), VanTC(1), VanTE(1), VanTG(1), VanTN(1), VanTmL(1), VanTrL(1), VanUG(1), VanVB(1), VanWB(1), VanWG(1), VanWI(1), VanXA(1), VanXB(1), VanXD(1), VanXE(1), VanXI(1), VanXM(1), VanXO(1), VanXYC(1), VanXYE(1), VanXYG(1), VanXYL(1), VanXYN(1), VanYA(1), VanYB(1), VanYD(1), VanYF(1), VanYG1(1), VanYM(1), VanZA(1), VanZF(1)
Macrolides		CarA(1), CfrA(1), CfrB(1), CfrC(1), Emt(1), EreA(2), EreB(1), EreD(1), Erm(47), LinG(1), LnuA(1), LnuB(1), LnuC(1), LnuD(1), LnuF(2), LnuG(1), LnuP(1), LsaA(1), LsaB(1), LsaC(1), LsaE(1), MefB(1), Mel(1), MgtA(1), MphA(1), MphB(2), MphC(2), MphE(2), MphF(1), MphG(1), MphH(1), MphI(1), MphJ(1), MphK(1), MphL(1), MphM(1), MphN(1), MphO(1), MsrA(2), MsrC(2), MsrE(1), MyrA(1), OleB(1), OleC(1), OleD(1), OleI(1), OptrA(1), PoxtA(1), SrmB(1), TirC(1), TvaA(1), VatA(1), VatB(1), VatC(1), VatD(2), VatE(1), VatF(1), VatH(1), VatI(1), VgaA(2), VgaB(1), VgaC(1), VgaD(1), VgaE(2), VgbA(1), VgbB(1), VgbC(1), VmlR(1), clbB(1), clbC(1), cleD(1), gimA(1)
Phenicols		Cat(33), CatBx(1), CmlA(5), CmlB(2), CmlR(1), CmlV(1), Cmr(1), CmrA(2), FloR(2), PexA(1)
Rifampin		Arr(7), IRI(1), RphB(1)
Sulfonamide		Sul(4)
Tetracycline		OtrA(1), OtrB(1), OtrC(1), Tcr3(1), Tet-30(1), Tet-31(1), Tet-32(2), Tet-33(1), Tet-35(1), Tet-36(1), Tet-37(1), Tet-38(1), Tet-39(1), Tet-40(1), Tet-41(1), Tet-42(1), Tet-43(1), Tet-44(1), Tet-45(1), Tet-48(1), Tet-49(1), Tet-50(1), Tet-51(1), Tet-52(1), Tet-53(1), Tet-54(1), Tet-55(1), Tet-56(1), Tet-59(1), TetA(7), TetB(6), TetC(1), TetD(1), TetE(2), TetG(2), TetH(2), TetJ(1), TetK(1), TetL(2), TetM(2), TetO(1), TetQ(1), TetS(1), TetT(1), TetU(1), TetV(1), TetW(2), TetX(1), TetY(1), TetZ(1)
Tigecycline		TetX(2), tmexC(1), tmexD(1), toprJ(1)
Trimethoprim		Dfr(52)

*Numbers in the last brackets are the number of alleles.

Table S4. Species supported in CCNE*.

Class	Group/Family	Genus	Species
Aerobic Gram-positive cocci	Staphlococcaceae	*Staphylococcus*	***Staphylococcus aureus*** *,* ***Staphylococcus epidermidis*** *, Staphylococcus haemolyticus,* ***Stapylococcus hominis*** *, Staphylococcus lugdunensis, Staphylococcus pseudintermedius*
	Streptococcaceae	*Streptococcus*	***Streptococcus agalactiae*** *, Streptococcus canis, Streptococcus dysgalactiae, Streptococcus gallolyticus, Streptococcus oralis,* ***Streptococcus pneumoniae*** *,* ***Streptococcus pyogenes*** *, Streptococcus suis, Streptococcus thermophilus, Streptococcus uberis, Streptococcus equi*
	Enterococcaceae	*Enterococcus*	***Enterococcus faecalis, Enterococcus faecium***
Aerobic Gram-positive bacillus	Listeriaceae	*Listeria*	*Listeria monocytogenes*
	Lactobacillaceae	*Lactobacillus*	*Lactobacillus salivarius*
	Bacillaceae	*Bacillus*	*Bacillus cereus, Bacillus licheniformis, Bacillus subtilis*
	Corynebacteriaceae	*Corynebacterium*	*Corynebacterium diphtheriae*
	Streptomycetaceae	*Streptomyces*	*Streptomyces spp.*
	Mycobacteriaceae	*Mycobacterium*	*Mycobacterium abscessus, Mycobacterium massiliense*
Aerobic Gram-negative cocci	Neisseriaceae	*Neisseria*	*Neisseria spp.*
	Moraxellaceae	*Moraxella*	*Moraxella catarrhalis*
Aerobic Gram-negative bacillus	Enterobacteriaceae	*Escherichia*	***Escherichia spp.***
		Shigella	*Shigella spp.*
		Salmonella	*Salmonella enterica*
		Yersinia	*Yersinia spp., Yersinia pseudotuberculosis, Yersinia ruckeri*
		Klebsiella	***Klebsiella oxytoca, Klebsiella pneumoniae, Klebsiella aerogenes***
		Enterobacter	***Enterobacter cloacae***
		Citrobacter	*Citrobacter freundii*
		Serratia	***Serratia marcescens***
		Proteus	***Proteus mirabilis***
		Edwardsiella	*Edwardsiella tarda*
Aerobic Gram-negative bacillus (Non-Enterobacteriaceae)	Aeromonadaceae	*Aeromonas*	*Aeromonas spp.*
	Vibrionaceae	*Vibrio*	*Vibrio cholerae, Vibrio spp., Vibrio parahaemolyticus, Vibrio tapetis, Vibrio vulnificus*
Aerobic Gram-negative bacillus (Sugar unfermented)	Pseudomonadaceae	*Pseudomonae*	***Pseudomonas aeruginosa*** *, Pseudomonas fluorescens*
	Moraxellaceae	*Acinetobacter*	***Acinetobacter baumannii***
	Flavobacteriaceae	*Flavobacterium*	*Flavobacterium psychrophilum*
	Burkholderiaceae	*Burkholderia*	***Burkholderia cepacia*** *, Burkholderia pseudomallei*
	Xanthomonadaceae	*Stenotrophomonas*	***Stenotrophomonas maltophilia***
	Alcaligenaceae	*Achromobacter*	*Achromobacter spp.*
Other aerobic Gram-negative bacteria	Pasteurellaceae	*Hemophilus*	***Haemophilus influenzae*** *, Haemophilus parasuis, Haematopinus suis*
	Neisseriaceae	*Kingella*	*Kingella kingae*
	Alcaligenaceae	*Bordetella*	*Bordetella pertussis*
	Pasteurellaceae	*Pasteurella*	*Pasteurella multocida*
	Bartonellaceae	*Bartonella*	*Bartonella henselae*
Anaerobic Gram-positive bacillus (Non-Spore-Forming)	Propionibacteriaceae	*Propionibacterium*	*Propionibacterium acnes*
Anaerobic Gram-positive bacillus (Spore-Forming)	Clostridiaceae	*Clostridium*	*Clostridium botulinum, Clostridium difficile, Clostridium septicum*
Anaerobic Gram-negativ bacillus	Porphyromonasaceae	*Porphyromonas*	*Porphyromonas gingivalis*
Gram-negative Campylobacter and Helicobacter	Campylobacteraceae	*Campylobacter*	*Campylobacter coli, Campylobacter jejuni, Campylobacter concisus, Campylobacter fetus, Campylobacter helveticus, Campylobacter hyointestinalis, Campylobacter insulaenigrae, Campylobacter lanienae, Campylobacter lari, Campylobacter sputorum, Campylobacter upsaliensis*
		Arcobacter	*Arcobacter spp.*
	Helicobacteraceae	*Helicobacte*	*Helicobacter cinaedi, Helicobacter pylori*
Spirochetes	Leptospiraceae	*Leptospira*	*Leptospira*
		Borrelia	*Borrelia spp.*
		Brachyspira	*Brachyspira hampsonii, Brachyspira hyodysenteriae, Brachyspira intermedia, Brachyspira pilosicoli, Brachyspira*
Mycoplasma, Chlamydia etc.	Mycoplasmataceae	*Mycoplasma*	*Mycoplasma agalactiae, Mycoplasma bovis, Mycoplasma hyorhinis*
	Chlamydiaceae	*Chlamydia*	*Chlamydia spp.*
	Rickettsiaceae	*Orientia*	*Orientia tsutsugamushi*
Other			*Anaplasma aphagocytophilum*
			Peptoclostridium difficile
			Carnobacterium maltaromaticum
			Cronobacter spp.
			Mannheimia haemolytica
			Melissococcus plutonius
			Ornithobacterium rhinotracheale
			Paenibacillus larvae
			Pediococcus pentosaceus
			Riemerella anatipestifer
			Sinorhizobium spp.
			Taylorella spp.
			Tenacibaculum spp.
			Wolbachia spp.
			Xylella fastidiosa

*Species in blod are most commonly isolated species in clinical. (Data come from the CHINET: http://www.chinets.com/Data/AntibioticDrugFast)

Table S5. The performance of all the methods in simulation.

Dataset	Measurement*	CCNE-acc	CCNE-fast	AssemblyBased	OrthologsBased
Without noise	Accurary	100%	96.30%	98.90%	99.30%
	RMSE	0.09	0.21	0.21	0.16
With noise	Accurary	100%	97.10%	23.40%	78.90%
	RMSE	0.07	0.21	1.7	0.39
Tandem repeat	Accurary	100%	95.80%	19.50%	99.90%
	RMSE	0.07	0.22	2.54	0.13
Mix	Accurary	100%	97.70%	10.50%	99.70%
	RMSE	0.07	0.2	1.89	0.14

*RMSE: root mean squard error

Table S6. The published data from GenBank used for estimating the copy numbers of blaKPC-2.

Species	Genomes in GenBank (n)	Genomes harboring blaKPC-2 (n)	Samples with SRA short reads (n)
K. pneumoniae	10,692	2,840	808
E. coli	23,447	127	46
P. aeruginosa	5,850	34	11
